# Are there subgroups of chronic fatigue syndrome? An exploratory cluster analysis of biological markers

**DOI:** 10.1186/s12967-021-02713-9

**Published:** 2021-01-30

**Authors:** Tarjei Tørre Asprusten, Line Sletner, Vegard Bruun Bratholm Wyller

**Affiliations:** 1 Department of Paediatric and Adolescent Health, Akershus University Hospital, 1478 Lørenskog, Norway; 2 Division of Medicine and Laboratory Sciences, University of Oslo, Oslo, Norway

**Keywords:** Chronic fatigue syndrome, Adolescent, Cluster analysis, Diagnostic criteria

## Abstract

**Background:**

Chronic fatigue syndrome (CFS) is defined according to subjective symptoms only, and several conflicting case definition exist. Previous research has discovered certain biological alterations. The aim of the present study was to explore possible subgroups based on biological markers within a widely defined cohort of adolescent CFS patients and investigate to what extent eventual subgroups are associated with other variables.

**Methods:**

The Norwegian Study of Chronic Fatigue Syndrome in Adolescents: Pathophysiology and Intervention Trial (NorCAPITAL) has previously performed detailed investigation of immunological, autonomic, neuroendocrine, cognitive and sensory processing functions in an adolescent group of CFS patients recruited according to wide diagnostic criteria. In the present study, hierarchical cluster analyses (Ward’s method) were performed using representative variables from all these domains. Associations between clusters and constitutional factors (including candidate genetic markers), diagnostic criteria, subjective symptoms and prognosis were explored by standard statistical methods.

**Results:**

A total of 116 patients (26.7% males, mean age 15.4 years) were included. The final cluster analyses revealed six clusters labelled pain tolerant & good cognitions, restored HPA dynamics, orthostatic intolerance, low-grade inflammation, pain intolerant & poor cognitions, and high vagal (parasympathetic) activity, respectively. There was substantial overlap between clusters. The pain intolerant & poor cognitions-cluster was associated with low functional abilities and quality of life, and adherence to the Canada 2003 diagnostic criteria for CFS. No other statistically significant cluster associations were discovered.

**Conclusion:**

Within a widely defined cohort of adolescent CFS patients, clusters could be delineated, but no distinct subgroups could be identified. Associations between clusters and constitutional factors, subjective symptoms and prognosis were scarce. These results question the clinical usefulness of searching for CFS subgroups, as well as the validity of the most “narrow” CFS diagnostic criteria.

*Trial registration*: Clinical Trials NCT01040429

## Background

Chronic Fatigue (CF) affects a substantial proportion of the population. In adolescents, about 20% of girls and 6.5% of boys report to have been severely fatigued during the last month [1, 2]. The label Chronic Fatigue Syndrome (CFS), sometimes referred to as Myalgic Encephalomyelitis (ME), may be appropriate if the fatigue is unexplained, long lasting, disabling and accompanied by other symptoms such as post exertional malaise, musculoskeletal pain, orthostatic intolerance, and cognitive problems [3]. Adolescent CFS prevalence is estimated at 0.1 to 1.0% [4–6], and CFS may have detrimental effects on psychosocial and academic development [7, 8], as well as family functioning [8].

The diagnostic criteria of CFS has been a scientific controversy for decades. As no diagnostic biomarker has been discovered, the diagnosis depends upon specific constellation of symptoms. One part of the scientific community has promoted wide diagnostic criteria [9–11], and also maintained that CFS is most properly understood as a variant belonging to an even broader category, such as Functional Somatic Syndrome [12] or Bodily Distress Syndrome [13]. This “lumping together” tendency has been strongly opposed by another part of the scientific community, advocating CFS as a heterogeneous group of patients with different diseases and pathophysiological features, e.g., ME is claimed as a distinct unique entity different from other fatiguing conditions, such as reflected in the Canadian diagnostic criteria of CFS (sometimes referred to as the International Consensus Criteria of ME/CFS) [14, 15]. The commonly used Fukuda-criteria [16] as well as the more recently proposed SEID-criteria [3] may be taken to represent pragmatic compromises.

Nevertheless, the net result has been a confusing existence of at least 20 case definitions. Most of them require between 3 and 6 months of unexplained fatigue but vary considerably regarding requirement of additional symptoms. In a systematic review from 2014, Brurberg et al. could not draw firm conclusions concerning the validity of any of these criteria due to weak methodology and inconsistent results of the 38 included validation studies [17]. Accordingly, studies from our own institution question the validity of the Canadian-criteria [18] as well as the SEID-criteria [19] for diagnosing CFS in adolescents.

In an attempt of investigating possible subgroups within widely defined CFS cohorts, latent class analyses have been applied; recent reports suggest the presence of discrete endophenotypes [20–23]. However, these approaches still rely on subjective reporting of symptoms, and it remains unclear to what extent a specific endophenotype corresponds with certain pathophysiological mechanisms or etiological factors. The presence or not of such correspondence may be considered essential for a proper understanding of the underlying disease mechanisms of CFS.

Despite the absence of diagnostic biomarkers, associations between CFS and candidate genetic markers as well as certain aberrations of immunological, autonomic, neuroendocrine, cognitive and sensory processing functions have been firmly established in previous research [24]. As for genetic markers, a single nucleotide polymorphisms (SNP) in the gene encoding the catecholaminergic breakdown enzyme COMT (catechol-O-methyltransferase) has been linked to CFS in several reports [25, 26]. In addition, mutations in the serotoninergic system are one of the most consistently reported findings in genetic studies of CFS [27–29]. As for immunological aberration, the most consistent finding appears to be a tendency towards low-grade systemic inflammation, as reflected in elevated serum C-reactive protein (CRP) [30], elevated pro-inflammatory cytokines [31, 32], and increased levels of innate immunity gene products in whole blood gene expression analyses [33]. Also, low-grade inflammation has been hypothesized as a common pathophysiological phenomenon across fatigue states in general [34]. As for autonomic aberrations, most studies suggest a sympathetic predominance, reflected in increased sympathetic cardiovascular activity [35–38], decreased parasympathetic (vagal) heart rate control [39], altered sympathetic thermoregulatory responses [39], and increased plasma and urine catecholamines [30, 40]. This sympathetic predominance may be the underlying cause of the Postural Orthostatic Intolerance Syndrome (POTS) phenomenon, which is frequently observed among CFS patients [3]. As for neuroendocrine aberrations, attenuated hypothalamus–pituitary–adrenal [HPA] axis dynamics is a consistent finding across adult and adolescent CFS studies [30, 41–44]. Interestingly, normalization of HPA responses may be associated with improvement of symptoms and functional disabilities [43, 45]. As for cognitive functions, previous research has provided evidence of aberrations in the domains of attention, memory and reaction time [46–48]. Studies specifically addressing executive functions in adolescent CFS patients have reported impaired interference control [49, 50], cognitive flexibility [51], and working memory [50, 52]. Finally, as for sensory processing functions, three studies have reported strongly reduced pressure pain thresholds [53–55], suggestive of central sensitization to afferent sensory stimuli [56]. Accordingly, functional brain imaging studies have demonstrated differences across CFS patients and healthy controls [57].

Thus, an alternative approach for delineating possible CFS subgroups would be to use the above-mentioned biological aberrations as a point of departure instead of subjective symptoms when performing subgroup-generating statistical analyses. To the best of our knowledge, such an approach is novel in the field of CFS. In the present study, we aimed to: a) Explore possible subgroups based on biological aberrations within a widely defined cohort of adolescent CFS patients; b) Investigate to what extent these subgroups are associated with constitutional factors (including genetic markers), diagnostic criteria, subjective symptoms and prognosis.

## Methods

### Study design and ethics

This study is part of the Norwegian Study of Chronic Fatigue Syndrome in Adolescents: Pathophysiology and Intervention Trial (NorCAPITAL) (ClinicalTrials ID: NCT01040429), which is a combined cross-sectional and randomized controlled trial of low-dose clonidine in adolescent CFS; the design has been described in detail elsewhere [30]. In the present study, we used baseline data and follow-up data from week 30, collected between March 2010 and October 2012. The study was approved by the Regional Committee for Medical and Health Research Ethics for South-East Norway and the Norwegian Medicines Agency and adhered to the Declaration of Helsinki. Informed, written consent was obtained from all participants and from parents or next-of-kin if required.

### Recruitment of CFS patients

All 20 hospital paediatric departments in Norway primary care paediatricians and general practitioners were invited to refer adolescents with CFS aged 12 to 18 years consecutively to the Department of Paediatrics at Oslo University Hospital, which served as a national referral center for young patients with CFS. To be eligible for the NorCAPITAL project, we required 3 months of unexplained chronic/relapsing fatigue of new onset. The patients were not required to meet any additional symptom criteria, in line with clinical Paediatric guidelines [9]. A standard form required the referral unit to confirm the result of clinical investigations considered compulsory to diagnose pediatric CFS (specialist evaluation, extensive hematology and biochemistry work-up, chest X-ray, abdominal ultrasound, and brain MRI). Also, the referring units were required to confirm that the patient (a) was hindered from normal school attendance due to fatigue; (b) was not permanently bedridden; (c) was not stroked by a medical or psychiatric disorder (including depression) and/or did not go through any concurrent demanding life event; and (d) did not use medicines (including hormone contraceptives) regularly. Patients considered eligible were summoned to our study center; a final decision on inclusion was made after a separate clinical examination combined with quality assessment of the previously conducted screening program. Details of the recruitment procedure and inclusion/exclusion criteria are described elsewhere [29].

All participants underwent an identical investigational program at baseline, 8 weeks and 30 weeks, which included a one day in-hospital assessment encompassing clinical examination, blood sampling, autonomic testing, and cognitive testing. Immediately afterwards, daily physical activity was monitored during seven consecutive days, and a self-administered questionnaire was completed.

### Markers of biological aberrations

All methods for assessing markers of biological aberrations have been thoroughly described in previous publications from the NorCAPITAL project [30, 45, 50, 55, 58, 59]; a brief description is provided below.

Immunological markers were investigated by examining plasma CRP level through a high-sensitive assay (Roche Diagnostics, Indianapolis, IN, USA), and by measuring 27 plasma cytokines, including interleukins, chemokines and growth factors, using a multiplex technique (Bio-Plex Human Cytokine 27-Plex; Bio-Rad Laboratories Inc., Hercules, CA, USA) [58].

Autonomic markers were investigated using the Task Force Monitor ^®^ (Model 3040i, CNSystems Medizintechnic, Graz, Austria), a combined hardware and software device for noninvasive continuous recording of autonomic cardiovascular control [60]. Supine values as well as responses to a low intensity 20 deg. head-up tilt test (HUT) are reported [59]. Power spectral analysis of heart rate variability (HRV) was calculated in the Low Frequency (LF) range (0.05 to 0.17 Hz), and High Frequency (HF) range (0.17 to 0.4 Hz) [61]. Vagal (parasympathetic) activity is the main contributor to HF variability, whereas both vagal and sympathetic activity contributes to LF variability.

Neuroendocrine markers included plasma and urine norepinephrine and epinephrine. These markers were assayed by high-performance liquid chromatography (HPLC) with a reversed-phase column and glassy carbon electrochemical detector (Antec, Leyden Deacade II SCC, Zoeterwoude, The Netherlands), using a commercial kit (Chromsystems, München, Germany) [62]. Urine free cortisol (non-conjugated cortisol) was assayed by solid phase competitive luminescence immunoassay (LIA) (type Immulite^®^ 2000, Siemens Healthcare Diagnostics, NY, USA) after extraction from the urine sample with ether [63]. Plasma cortisol, adrenocorticotrophic hormone (ACTH), thyroid-stimulating hormone (TSH), and free thyroxine (FT4), as well as serum Insulin-like Growth Factor 1 (IGF1), were determined by routine assays at the accredited Hormone laboratory at Oslo University Hospital, Norway.

Cognitive function was assessed using the digit span test from the Wechsler Intelligence Scale for Children, 4th edition (WISC-IV) [64], the conditions 1–3 of Color-Word Interference test from the Delis-Kaplan Executive Function System (D-KEFS) [65], and the Total recall part of Hopkins Verbal Learning Test-Revised (HVLT-R) [66].

Pressure pain threshold was assessed by gradually applying increasing pressure to six predefined areas (the third finger’s cuticles, the trapezius muscle and the supraspinatus muscle bilaterally), by using the force transducer Commander™ Algometer, which has a rubber tip of 0.5 cm^2^ (JTECH Medical, Midvale, USA) [54]. Participants were asked to indicate the first sensation of pain during increasing pressure. All sites were assessed in the same order for each patient, and the pressure stimuli were applied twice to each spot and then averaged. Values were reported in Newton (N).

### Genotyping

Procedures for genotyping in the NorCAPITAL project have been described in detail elsewhere [29]. In short, genomic DNA was extracted from whole blood samples. Single Nucleotide Polymorphism (SNP) genotyping was carried out using custom TaqMan SNP genotyping assays (Applied Biosystems, Foster City, CA, USA). Approximately 10% of the samples were re-genotyped and the concordance rate was 100%. To determine the length of the polymorphic promoter region of the serotonin transporter (5-HTT)-gene (*SLC6A4*), the DNA sequence was first amplified by polymerase chain reaction (PCR) and then separated by gel electrophoresis. The PCR yielded a long (529 bp) and a shorter (486 bp) fragment [67]. After four hours separation at 100 V on a 2.5% agarose gel (MetaPhor Agarose, Lonza cologne GmbH, Cologne, Germany), GelRed dye was added and the fragments were visualized by UV light (Biotium Inc, California, USA). The PCR 100 bp low ladder (Sigma-Aldrich CO, St. Louis, Mo, USA) was used to determine the length of the fragments.

### Questionnaires

A CFS symptom inventory for adults [68] has previously been used to develop an analogous inventory for adolescents [30]. A total of 24 common symptoms are evaluated in terms of frequency during the last month (five-point Likert scale ranging from never/rarer than once a month to present every day/almost every day, scored from 1 to 5). The questionnaire includes case defining symptoms of CFS according to the Canada as well as the Fukuda definition. As a general rule, all symptoms required in the definitions had to be present more than once a week (corresponding to a score of three or higher) for patients to be categorized as CFS [18].

In addition, validated inventories were used to assess symptoms and disabilities.

Fatigue was assessed by the Chalder Fatigue Questionnaire (CFQ), which encompasses 11 items scored on a 4-point (0 to 3) Likert scale [69]; total sum score is applied. Depressive symptoms were charted with the Mood and Feelings Questionnaire (MFQ) consisting of 34 items scored on a 3-point (0 to 2) Likert scale [70]; total sum score is applied. Quality of life was assessed with the Pediatric Quality of Life Inventory (PedsQL), consisting of 23 items scored on a 5-point (0-25-50-75-100) Likert scale [71]; mean score across all items is applied. Functional disability was assessed using the Functional Disability Inventory (FDI) encompassing 15 items scored on a 5-point (0-4) Likert scale [72]; total sum score is applied.

Finally, the symptom of post-exertional malaise (PEM) was charted by a single item: “How often do you experience more fatigue the day after an exertion?”, scored on a 5-point [1–5] Likert scale.

### Daily physical activity

The activPAL accelerometer device (PAL Technologies Ltd, Glasgow, Scotland) was used to provide data on step number and cadence as well as time spent on walking, standing and sitting/lying during everyday activities [73]. A recording period of seven consecutive days was selected. For each participant, all recording epochs were carefully and independently reviewed, and the mean number of steps per day was calculated for all recording epochs. Details on the activity recording procedure have been reported elsewhere [30].

### Cluster construction

**Fig. 1:**
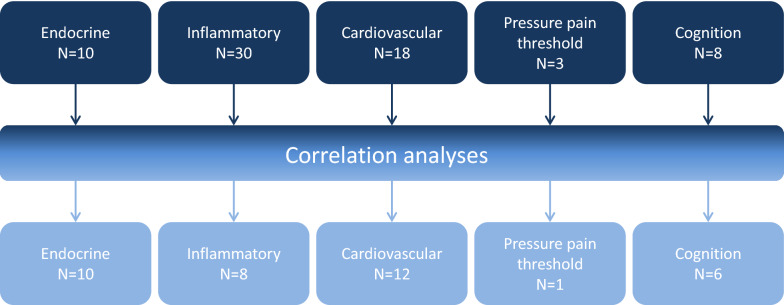
Number of variables before and after correlation analyses. A total of 69 different biomarkers was selected from the NorCAPITAL database and grouped into five domains (upper row): Endocrine, inflammatory, cardiovascular, pressure pain threshold, and cognitions. Correlation analyses among variables within each domain were performed. When two or more variables were strongly correlated (correlation coefficient ≥ 0.7), the variable considered most suitable for further analyses was carried over. Then, a final correlation analysis of all remaining variables from each domain were performed, resulting in a total of 37 variables across each of the five groups (lower row) which become the basis for subsequent cluster analyses

A total of 69 different biomarkers was selected from the NorCAPITAL database for analyses in the present study; the selection was guided by expert knowledge of the CFS/ME scientific literature. The biomarkers were grouped into five domains: endocrine (n = 10), inflammatory (n = 30), cardiovascular (n = 18), pressure pain threshold (n = 3), and cognitions (n = 8) (Fig. 1). Thereafter, in order to reduce the number of variables, correlation analyses among variables under each domain were performed. When two or more variables were strongly correlated (correlation coefficient ≥ 0.7), interpretability, suitability regarding statistical analyses and the size of the correlation coefficient were evaluated. The variable in total considered most suitable was kept for further analyses. A final correlation analysis of all remaining variables from each domain were performed, resulting in a total of 37 variables which become the basis for subsequent cluster analyses (Fig. 1, Additional file 1).

Firstly, hierarchical clustering analyses were performed within each of the five domains separately, using Ward’s method, squared Euclidian distance and Z-score. Thereafter, 1-3 variables from each domain were used for a final cluster analysis across all domains [74]. Variables were selected due to their importance in the cluster formation under each domain. The final number of clusters was decided primarily by visual inspection of dendrograms, but a preliminary validation of the possible cluster-solutions was also performed to ensure that there were meaningful differences between the clusters.

### Cluster validation

Associations between clusters and simple demographic variables, constitutional factors (including genetic markers) and adherence to CFS diagnostic criteria were explored (Table 3). Baseline values of CFQ, PEM, MFQ, Steps per day, PedsQL and FDI were used to investigate associations between clusters and markers of symptoms and function. Changes in markers of symptoms and function from baseline to week 30 were used to assess prognostic value of clusters.

Generally, differences across clusters were analyzed by Fisher’s exact test, one-way ANOVA or Kruskal–Wallis test as appropriate. All statistical analyses were carried out by SPSS statistical software. A p-value of < 0.05 was considered statistically significant. No correction for multiple testing was performed due to the exploratory nature of the analyses.

## Results

Of the 120 CFS/ME patients included in the NorCAPITAL project, four were excluded from further analyses due to lack of valid data, leaving 116 for analyses in the present study. In this group, 28% were males, and mean age was 15.4 years (Table 1).

**Table 1:** Background characteristics

Total number of participants	116
Sex-no. of males (%)	31 (26.7)
Age, years-mean (SD)	15.4 (1.6)
BMI, kg/m^2^-mean (SD)	21.5 (4.2)
Disease duration, months-mean (SD)	21.2 (14.7)
Fukuda-no. fulfilling criteria (%)^a^	85 (75.2)
Canada-no. fulfilling criteria (%)^b^	46 (41.4)

BMI: Body mass index; SD: Standard deviation

^a^Number of patients fulfilling the CDC Fukuda 1994 diagnostic criteria for Chronic Fatigue Syndrome [Fukuda 1994]

^b^Number of patients fulfilling the Canada 2003 diagnostic criteria for Chronic Fatigue Syndrome [14]

Separate cluster analyses within each domain of variables revealed substantial cluster overlap as well as few statistically significant associations with symptoms, functional abilities and prognosis. However, from each analysis it was possible to identify the most important variables driving the cluster formation, which in turn were carried over to the final cluster analysis across all domains (Additional file 2).

**Fig. 2:**
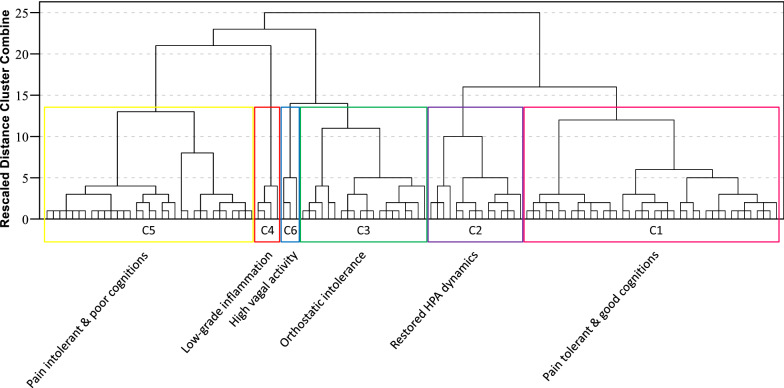
Final cluster solution and cluster characterizations. Dendrogram displaying the final cluster solution, revealing six clusters based on a total of 10 variables (each vertical bar on the x-axis corresponds to one individual). The clusters were labelled according to results presented in Table 2 (cf. manuscript for further details): The *pain tolerant & good cognitions*-cluster (C1) is characterized by high pressure pain threshold levels and high scores on cognitive function tests. The *restored HPA dynamics*-cluster (C2) is characterized by high urine cortisol:creatinin ratio. The *orthostatic intolerance*-cluster (C3) is characterized by a strong tachycardia response and corresponding fall in stroke volume during orthostatic challenge. The *low*-*grade inflammation*-cluster (C4) is characterized by high levels of interferon gamma (INFγ) and interferon gamma-induced protein 10 (IP-10). The *pain intolerant & poor cognitions*-cluster (C5) is characterized by low pressure pain threshold levels and low scores on cognitive function tests. The *high vagal activity*-cluster (C6) is characterized by high heart rate variability within the high-frequency (HF) domain

The final cluster solution revealed six clusters based on a total of 10 variables (Fig. 2, Table 2). Cluster 1 is characterized by high pressure pain threshold levels and high scores on cognitive function tests, and was labelled *pain tolerant & good cognitions.* Cluster 2 is characterized by high urine cortisol:creatinin ratio, which signalizes *restored HPA dynamics.* Cluster 3 is characterized by a strong tachycardia response and corresponding fall in stroke volume during orthostatic challenge, typical of *orthostatic intolerance.* Cluster 4 is characterized by high levels of interferon gamma (INFγ) and interferon gamma-induced protein 10 (IP-10), indicative of *low*-*grade inflammation.* Cluster 5 is characterized by low pressure pain threshold levels and low scores on cognitive function tests; this “mirror image” of cluster one is labelled *pain intolerant & poor cognitions.* Cluster 6 is characterized by strong power of heart rate variability within the high-frequency (HF) domain, reflecting *high vagal (parasympathetic) activity.* Few individuals (a total of 4 and 3, respectively) belonged to cluster 4 and cluster 6.

**Table 2:** Final cluster solution-contributing variables

	1: Pain tolerant & good cognitions (n = 40)	2: Restored HPA dynamics (n = 15)	3: Orthostatic intolerance (n = 20)	4: Low-grade inflammation (n = 4)	5: Pain intolerant & poor cognitions (n = 33)	6: High vagal activity (n = 3)
Plasma cortisol, nmol/L-mean (SD)	329 (118)	314 (132)	366 (132)	407 (136)	443 (154)	462 (169)
Urine cortisol:creatinine ratio, nmol/mmol-median (IQR)	3.2 (2.2, 4.4)	*14.5 (7.0, 18.4)*	3.2 (2.5, 4.9)	3.9 (2.8, 6.2)	3.2 (1.8, 4.4)	3.7 (n.a)
Interferon gamma, pg/mL-median (IQR)	84 (57, 119)	102 (63, 199)	125 (92, 250)	*174 (79, 223)*	65 (45, 116)	167 (n.a)
IP-10, pg/mL-median (IQR)	345 (261, 479)	419 (225, 467)	314 (323, 410)	*2735 (2359, 3803)*	313 (174, 502)	557 (n.a.)
HF-HRV supine, ms^2^-median (IQR)	1264 (422, 3262)	1743 (147, 2630)	869 (526, 2209)	101 (52, 126)	588 (198, 868)	*11052 (n.a.)*
Δ HR orthostatic response, beats/min-mean (SD)	3.5 (3.2)	2.5 (4.2)	*9.9 (4.1)*	3.9 (2.6)	4.7 (3.7)	7.8 (6.3)
Δ SI orthostatic response, mL/m^2^-mean (SD)	− 4.3 (2.9)	− 3.0 (5.6)	*− 11.4 (4.1)*	− 2.8 (5.4)	− 4.3 (3.3)	− 9.4 (5.0)
Pressure pain threshold, N/cm^2^-mean (SD)	*20.1 (7.6)*	16.5 (6.9)	15.2 (5.2)	13.2 (5.7)	*9.8 (3.9)*	17.1 (7.1)
Digit span forward, total score-mean (SD)	*9.3 (2.1)*	8.7 (1.5)	8.0 (2.1)	8.5 (2.4)	*7.2 (1.2)*	8.0 (1.0)
Color-Word interference test condition 2, sec-mean (SD)	25.7 (4.2)	22.9 (4.5)	25.9 (4.4)	25.3 (4.3)	*30.2 (9.2)*	23.7 (4.2)

HPA: hypothalamus–pituitary–adrenal; CI: confidence interval; IP-10: Interferon gamma-induced protein 10; HR: heart rate; SI: stroke index; HF-HRV: high-frequency power of heart rate variability; IQR: interquartile range (25 and 75 percentile). Δ denotes the response (upright–supine) to orthostatic challenge. The characterizing variable scores within each cluster is highlighted with italics

There were no significant differences between clusters regarding demographic and constitutional variables, including candidate genetic markers (Table 3). However, individuals belonging to Cluster 5—*Pain intolerant & poor cognitions*—were significantly more prone to adhere to the Canada 2003 diagnostic criteria for CFS. Also, this cluster had significantly poorer scores on the FDI and PedsQL functional inventories as compared to clusters 1–3 (Table 4). Symptoms scores for fatigue (CFQ), post-exertional malaise and depressive thoughts (MFQ) did not differ significantly across clusters. As for changes in symptoms and function over a 30-week follow-up period, there were no significant differences between the clusters, but a non-significant tendency for stronger functional improvement among Cluster 5—*Pain intolerant & poor cognitions* (Table 5).

**Table 3:** Final cluster solution–demographic, genetic and CFS criteria adherence differences

Cluster	1: Pain tolerant & good cognitions (n = 40)	2: Restored HPA dynamics (n = 15)	3: Orthostatic intolerance (n = 20)	4: Low-grade inflammation (n = 4)	5: Pain intolerant & poor cognitions (n = 33)	6: High vagal activity (n = 3)	p-value^c^
Sex-no. of males (%)	7 (17.5)	4 (26.7)	7 (35.0)	1 (25.0)	10 (30.3)	1 (33.3)	0.639
Age, years-mean [95% CI]	15.7 [15.3, 16.3]	15.7 [14.9, 16.5]	15.0 [14.2, 15.7]	16.5 [15.1, 18.0]	14.8 [14.3, 15.4]	15.4 [11.2, 19.6]	0.074
BMI, kg/m^2^-mean [95% CI]	22.3 [21.0, 23.1]	23.2[20.4, 26.1]	20.4 [17.9, 22.6]	22.8 [11.6, 33.9]	20.7 [19.4, 22.0]	18.3 [11.4, 25.1]	0.128
Disease duration, months-mean [95% CI]	19.4 [15.9, 22.9]	25.1 [10.8, 39.5]	22.0 [16.0, 27.9]	28.0 [n.a.]	19.3 [14.8, 23.9]	30.3 [n.a.]	0.538
Fukuda-no. fulfilling criteria (%)^a^	27 (71.1)	9 (60.0)	16 (80.0)	4 (100.0)	25 (78.1)	3 (100.0)	0.564
Canada-no. fulfilling criteria (%)^b^	13 (35.1)	3 (20.0)	6 (30.0)	1 (25.0)	22 (68.8)	1 (33.3)	0.006
COMT [SNP rs4680]-no. (%)							
AA	10 (25.0)	4 (26.7)	8 (40.0)	1 (25.0)	13 (39.4)	0 (0)	0.616
AG/GG	30 (75.0)	11 (73.3)	12 (60.0)	3 (75.0)	20 (60.6)	3 (100.0)	
SLC6A4 [5-HTTLPR allele & SNP rs25531]-no. (%)							
SS/SLG	11 (27.5)	3 (20.0)	2 (10.0)	3 (75.0)	9 (27.3)	1 (66.7)	0.139
SA/LALG/LALA	29 (27.5)	20 (80.0)	18 (90.0)	1 (25.0)	24 (72.7)	2 (33.3)	

HPA: hypothalamus–pituitary–adrenal; BMI: Body mass index; SD: Standard deviation; ADRA2A: alpha-2A adrenergic receptor gene; COMT: Catechol-O-methyltransferase gene; ADRB2: beta-2 adrenergic receptor gene; SLC6A4: serotonin transporter (5-HTT) gene; C: Cytosine; G: Guanine; A: Adenosine; LPR: long tandem repeats; S: short allele; L: long allele

^a^Number of patients fulfilling the CDC Fukuda 1994 diagnostic criteria for Chronic Fatigue Syndrome [Fukuda 1994]

^b^Number of patients fulfilling the Canada 2003 diagnostic criteria for Chronic Fatigue Syndrome [Carruthers 2003]

^c^Unadjusted p-values. The p-values are based on the Fischer exact test or one-way ANOVA, as appropriate. Only group 1–3 and 5 were used in the statistical analyses of continuous variables due to few participants in group 4 and 6

**Table 4:** Final cluster solution–differences in symptoms and functional abilities

Cluster	1: Pain tolerant & good cognitions (n = 40)	2: Restored HPA dynamics (n = 15)	3: Orthostatic intolerance (n = 20)	4: Low-grade inflammation (n = 4)	5: Pain intolerant & poor cognitions (n = 33)	6: High vagal activity (n = 3)	p-value^a^
CFQ-mean [95% CI]	18.9 [17.0, 20.9]	17.5 [13.8, 21.2]	18.6 [16.0, 21.1]	23.8 [19.0, 28.5]	20.7 [18.3, 23.1]	17.3 [10.2, 24.5]	0.344
PEM-mean (Mean Rank)	3.8 (47.9)	4.0 (56.6)	3.8 (48.1)	3.8 (n.a)	4.2 (59.0)	4.3 (n.a.)	0.074
MFQ-mean [95% CI]	18.1 [15.0, 21.2]	18.9 [10.6, 27.3]	14.7 [11.3, 18.0]	24.0 [12.5, 35.5]	17.9 [14.4, 21.4]	14.0 [-8.8, 36.8]	0.559
Steps per day-mean [95% CI]	5100 [4396, 5804]	5573 [3906, 7240]	4225 [3091, 5358]]	4015 [130, 7899]	3942 [3211, 4672]	3008 [1837, 4178]	0.063
FDI-mean [95% CI]	21.6 [19.2, 24.1]	19.7 [14.6, 24.7]	21.1 [17.2, 25.0]	28.8 [11.2, 46.3]	29.3 [25.8, 32.7]	21.0 [8.6, 33.4]	<0.001
PedsQL–mean [95% CI]	52.1 [48.5, 55.6]	51.7 [42.4, 60.9]	51.0 [44.6, 57.5]	44.0 [36.9, 51.1]	42.7 [38.2, 47.1]	44.5 [23.5, 65.6]	0.013

HPA: hypothalamus–pituitary–adrenal; SD: standard deviation; CFQ: Chalder Fatigue Questionnaire; PEM: Post Exertional Malaise; MFQ: Moods and Feelings Questionnaire; FDI: Funtion and Disability Inventory; PedsQL: Pediatric Quality of Life. n.a.: not applicable.

^a^Unadjusted p-values. The p-values are based on one-way ANOVA or Kruskal–Wallis test, as appropriate. Only group 1–3 and 5 were used in the statistical analyses due to few participants in group 4 and 6

**Table 5:** Final cluster solution–differences in development of symptoms and functions over time (baseline to week 30 follow-up)

Cluster	1: Pain tolerant & good cognitions (n = 40)	2: Restored HPA dynamics (n = 15)	3: Orthostatic intolerance (n = 20)	4: Low-grade inflammation (n = 4)	5: Pain intolerant & poor cognitions (n = 33)	6: High vagal activity (n = 3)	p-value^a^
Δ CFQ-mean [95% CI]	− 5.0 [− 7.6, − 2.4]	− 5.0 [− 10.5, 0.5]	− 4.8 [− 7.6, − 2.0]	− 6.5 [n.a]	− 5.2 [− 8.1, − 2.2]	4.0 [n.a.]	0.999
Δ PEM-mean [95% CI]	− 0.48 [− 0.9, 0.0]	− 0.36 [− 1.4, 0.6]	0.12 [− 0.2, 0.4]	− 1.00 [n.a.]	− 0.73 [− 1.2, − 0.3]	− 1.0 [n.a.]	0.130
Δ MFQ-mean [95% CI]	− 1.0 [− 4.1, 2.1]	− 1.7 [− 7.4, 3.9]	− 0.2 [− 3.6, 3.1]	− 4.7 [n.a.]	− 3.1 [− 6.8, 0.5]	− 3.0 [n.a.]	0.685
Δ Steps per day-mean [95% CI]	526 [− 593, 1644]	− 1107 [− 3004, 789]	− 578 [− 1506, 350]	1228 [n.a.]	283 [− 532, 1099]	− 657 [n.a.]	0.218
Δ FDI-mean [95% CI]	− 3.1 [− 6.2, 0.1]	− 1.9 [− 8.2, 4.4]	0.6 [− 3.4, 4.6]	− 13.0 [n.a.]	− 6.7 [− 10.8, − 2.5]	− 9.5 [n.a.]	0.079
Δ PedsQL-mean [95% CI]	6.6 [0.8, 12.5]	5.8 [− 6.4, 18.1]	2.5 [− 2.7, 7.7]	6.9 [n.a.]	8.4 [4.3, 12.5]	14.2 [n.a.]	0.584

HPA: hypothalamus–pituitary–adrenal; CI: Confidence Interval; n.a.: not applicable; CFQ: Chalder Fatigue Questionnaire; PEM: Post Exertional Malaise; MFQ: Moods and Feelings Questionnaire; FDI: Funtion and Disability Inventory; PedsQL: Pediatric Quality of Life

^a^Unadjusted p-values. The p-values are based on one-way ANOVA or Kruskal–Wallis test, as appropriate. Only group 1–3 and 5 were used in the statistical analyses due to few participants in group 4 and 6

**Fig. 3:**
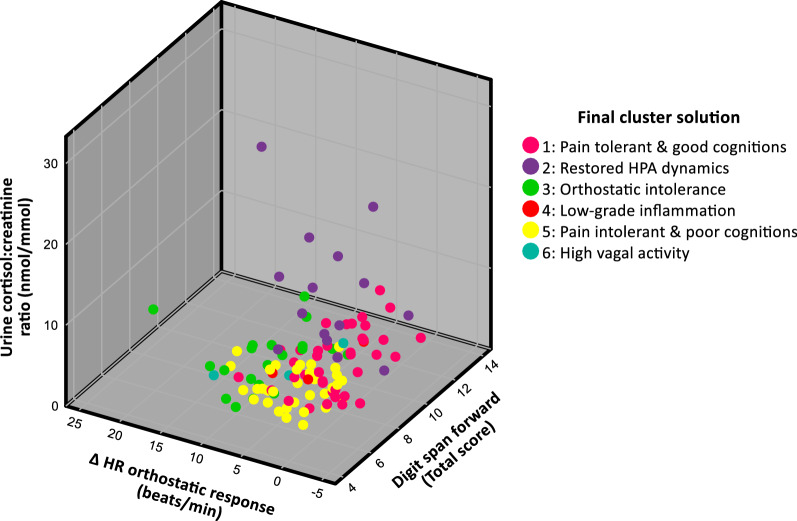
Scatterplot of the three most important variables for the final cluster formation. Each colored dot represents one individual belonging to one of the six clusters from the final cluster solution. Even though the three most important variables driving the cluster formation are used as coordinates, there is substantial overlap between the clusters

A scatterplot of the three most important variables for the final cluster formation (urine cortisol:creatinine ratio, Δ HR orthostatic response and digit span forward) revealed a substantial overlap between the clusters (Fig. 3).

## Discussion

The most important finding of the present study is that within a widely defined cohort of adolescent CFS patients, clusters corresponding to certain pathophysiological characteristics could be delineated, but overlap between clusters were substantial and no distinct subgroups could be identified. Also, there were scarce associations between clusters and constitutional factors, subjective symptoms and prognosis.

Medical diagnoses remain the foundations for treatment, rehabilitation, and prognostic assumptions; hence, the importance of valid diagnostic entities can hardly be underestimated. Sadly, the lack of objective criteria for CFS has contributed to the co-existence of multiple sets of diagnostic criteria, all of which are based on subjective reporting of symptoms. It is frequently maintained that certain case definitions correspond to specific underlying disease mechanisms; for instance, the Canada 2003 definition put strong emphasis on a possible inflammatory pathophysiology [14]. Accordingly, it is often argued that studies based on a wide diagnostic definition of CFS (i.e., a definition that requires a minimum of accompanying symptoms), such as the Oxford criteria [11], are at risk of introducing substantial heterogeneity in the patient sample which in turn may obscure results that pertain to a specific subgroup only. Specifically, it is frequently maintained that the use of a wide diagnostic definition in clinical trials tend to select a large portion of patients suffering from mental distress who may benefit from psychological/behavioural interventions, whereas such interventions are claimed to be unhelpful (or even harmful) for the potential subgroup of patients suffering from another (such as inflammatory) disease mechanism [75].

This argument seems to rely upon an a priori-assumption of the existence of subgroups within a widely defined CFS cohort, and the related research efforts tend to focus on how such subgroups can be found from analysis of patients’ symptoms (or biomarkers yet to be discovered). However, a more fundamental scientific question, which has scarcely been addressed in previous research, is whether such subgroups exist at all. In the present study, while the cluster analyses did suggest some delineation corresponding to previously identified characteristics of CFS pathophysiology, such as low-grad inflammation, altered HPA dynamics, and orthostatic intolerance, the most striking finding is the absence of well-defined subgroups. Rather, the data seems to represent continuous variables in a multidimensional space. Accordingly, the clusters were not significantly associated with symptom scores nor prognosis. Taken together, the findings of the present paper favor a “lumping together” rather than “splitting apart” approach to CFS caseness, and question the clinical usefulness of searching for CFS subgroups as well as the validity of the most “narrow” CFS diagnostic criteria. If confirmed by future research, this finding may have important clinical implications. It would suggest, for instance, that well-documented rehabilitation strategies might be applicable to a wide range of CFS sufferers.

That said, the cluster analysis did reveal some interesting associations, such as the positive association between restored HPA axis and functional abilities, confirming findings from previous reports [43, 45]. Also, there was an association between low pain tolerance and cognitive functions, poor functional abilities and quality of life, and adherence to the Canada 2003 diagnostic definition of CFS [14]. The causality of these associations remains to be clarified; for instance, functional disability may have a negative impact on cognitive test performance, as well as the other way round. Anyway, a better characterization of this particularly vulnerable group of CFS patients may help to tailor clinical rehabilitation programs.

Interestingly, while low-grade inflammation is advocated as an important pathophysiological feature of CFS patients adhering to the Canada 2003 diagnostic criteria [14], results from the present study opposes these assumptions. The cluster characterized by low-grade inflammation was not associated with the Canada 2003 case definition for CFS, while the cluster characterized as *Pain intolerant & poor cognitions,* which actually was associated with the Canada 2003 case definition, had the lowest score on inflammation variables. This result corroborates previous finding from our group [18], and further questions the validity of the Canada 2003 case definition.

### Study strengths and limitations

A strength of the present study is the detailed characterization of CFS pathophysiology within several domains. Limitations include the relatively low number of CFS patients, leaving some of the clusters with few participants, and the study should therefore be regarded exploratory. Also, the study included adolescent patients only, and it is unknown to what extent results can be generalized to adults. Further research should seek to validate the present findings in a larger cohort of adult CFS patients.

The question on how to measure fatigue is a controversy in the field of CFS. The present study assumed a priori that fatigue is best conceptualized as a subjective sensation [76]; accordingly, a validated instrument based on self report (the Chalder Fatigue Questionnaire) was selected to operationalize fatigue. We acknowledge, however, that other researchers maintain that fatigue should be measured by objective standards (e.g. activity recordings). Also, recent findings suggest that the symptom of post-exertional malaise (PEM) is even more central to the phenomenon of CFS than previously understood, and that it should be assessed with comprehensive, validated instruments [77]. Unfortunately, these instruments were not available when the present study was planned.

## Conclusion

Within a widely defined cohort of adolescent CFS patients, clusters could be delineated based on biological markers, but no distinct subgroups could be identified. Associations between clusters and constitutional factors, subjective symptoms and prognosis were scarce. These results question the clinical usefulness of searching for CFS subgroups, as well as the validity of the most “narrow” CFS diagnostic criteria.

## Supplementary information

**Additional file 1.** Initial correlation analyses of all variables considered for hierarchical cluster analysis.

**Additional file 2.** Results of hierarchical cluster analyses within each subdomain of variables (immunological, autonomic, neuroendocrine, cognitive and sensory processing functions), as well as associations between clusters and constitutional factors, diagnostic criteria, subjective symptoms and prognosis.

## Data Availability

The dataset generated and analyzed during the current study is available from the corresponding author upon reasonable request.

## References

[1] Pawlikowska T, Chalder T, Hirsch SR, Wallace P, Wright DJ, Wessely SC (1994). Population based study of fatigue and psychological distress. BMJ.

[2] Crawley E (2014). The epidemiology of chronic fatigue syndrome/myalgic encephalitis in children. Arch Dis Child.

[3] IOM (Institute of Medicine). Beyond Myalgic Encephalomyelitis/Chronic Fatigue Syndrome: Redefining an Illness. Washington, DC: The National Academies Press; 2015. http://www.iom.edu/mecfs. 25695122

[4] Crawley EM, Emond AM, Sterne JAC (2011). Unidentified Chronic Fatigue Syndrome/myalgic encephalomyelitis (CFS/ME) is a major cause of school absence: surveillance outcomes from school-based clinics. BMJ Open.

[5] Jason L, Jordan K, Miike T, Bell D, Lapp C, Torres-Harding S (2006). A pediatric case definition for myalgic Encephalomyelitis and chronic fatigue syndrome. J Chronic Fatigue Syndr..

[6] Nijhof SL, Maijer K, Bleijenberg G, Uiterwaal CSPM, Kimpen JLL, van de Putte EM (2011). Adolescent chronic fatigue syndrome: prevalence, incidence, and morbidity. Pediatrics.

[7] Kennedy G, Underwood C, Belch JJ (2010). Physical and functional impact of chronic fatigue syndrome/myalgic encephalomyelitis in childhood. Pediatrics.

[8] Missen A, Hollingworth W, Eaton N, Crawley E (2012). The financial and psychological impacts on mothers of children with chronic fatigue syndrome (CFS/ME). Child Care Health Dev.

[9] Health RCoPaC. Evidence Based Guideline for the Management of CFS/ME (Chronic Fatigue Syndrome/Myalgic Encephalopathy) in Children and Young People. Royal College of Paediatrics and Child Health. 2004.

[10] NICE. Chronic Fatigue Syndrome/Myalgic Encephalomyelitis (or Encephalopathy): Diagnosis and Management of Chronic Fatigue Syndrome/Myalgic Encephalomyelitis (or Encephalopathy) in Adults and Children. London, Excellence NIfHaC. 2007. 21563329

[11] Sharpe MC, Archard LC, Banatvala JE, Borysiewicz LK, Clare AW, David A (1991). A report–chronic fatigue syndrome: guidelines for research. J R Soc Med.

[12] Wessely S, Nimnuan C, Sharpe M (1999). Functional somatic syndromes: one or many?. Lancet.

[13] Petersen MW, Schröder A, Jørgensen T, Ørnbøl E, Dantoft TM, Eliasen M (2020). The unifying diagnostic construct of bodily distress syndrome (BDS) was confirmed in the general population. J Psychosom Res.

[14] Carruthers BM, Jain AK, De Meirleir KL, Peterson DL, Klimas NG, Lerner AM (2003). Myalgic Encephalomyelitis/chronic fatigue syndrome: clinical working case definition, diagnostic and treatment protocols. J Chronic Fatigue Syndr..

[15] Carruthers BM, van de Sande MI, De Meirleir KL, Klimas NG, Broderick G, Mitchell T (2011). Myalgic encephalomyelitis: international consensus criteria. J Intern Med.

[16] Fukuda K, Straus SE, Hickie I, Sharpe MC, Dobbins JG, Komaroff A (1994). The chronic fatigue syndrome: a comprehensive approach to its definition and study. International Chronic Fatigue Syndrome Study Group. Ann Intern Med.

[17] Brurberg KG, Fonhus MS, Larun L, Flottorp S, Malterud K (2014). Case definitions for chronic fatigue syndrome/myalgic encephalomyelitis (CFS/ME): a systematic review. BMJ Open.

[18] Asprusten TT, Fagermoen E, Sulheim D, Skovlund E, Sorensen O, Mollnes TE (2015). Study findings challenge the content validity of the Canadian Consensus Criteria for adolescent chronic fatigue syndrome. Acta Paediatr.

[19] Asprusten TT, Sulheim D, Fagermoen E, Winger A, Skovlund E, Wyller VB (2018). Systemic exertion intolerance disease diagnostic criteria applied on an adolescent chronic fatigue syndrome cohort: evaluation of subgroup differences and prognostic utility. BMJ Paediatr Open..

[20] Huber KA, Sunnquist M, Jason LA (2018). Latent class analysis of a heterogeneous international sample of patients with myalgic encephalomyelitis/chronic fatigue syndrome. Fatigue Biome Health Behav..

[21] Williams TE, Chalder T, Sharpe M, White PD (2017). Heterogeneity in chronic fatigue syndrome-empirically defined subgroups from the PACE trial. Psychol Med.

[22] Aslakson E, Vollmer-Conna U, White PD (2006). The validity of an empirical delineation of heterogeneity in chronic unexplained fatigue. Pharmacogenomics..

[23] Aslakson E, Vollmer-Conna U, Reeves WC, White PD (2009). Replication of an empirical approach to delineate the heterogeneity of chronic unexplained fatigue. Popul Health Metrics..

[24] Rivera MC, Mastronardi C, Silva-Aldena CT, Arcos-Burgos M, Lidbury BA (2019). Myalgic encephalomyelitis/chronic fatigue syndrome: a comprehensive review. Diagnostics.

[25] Goertzel BN, Pennachin C, de Souza Coelho L, Gurbaxani B, Maloney EM, Jones JF (2006). Combinations of single nucleotide polymorphisms in neuroendocrine effector and receptor genes predict chronic fatigue syndrome. Pharmacogenomics..

[26] Sommerfeldt L, Portilla H, Jacobsen L, Gjerstad J, Wyller VB (2011). Polymorphisms of adrenergic cardiovascular control genes are associated with adolescent chronic fatigue syndrome. Acta Paediatr.

[27] Wang T, Yin J, Miller AH, Xiao C (2017). A systematic review of the association between fatigue and genetic polymorphisms. Brain Behav Immun.

[28] Smith AK, Dimulescu I, Falkenberg VR, Narasimhan S, Heim C, Vernon SD (2008). Genetic evaluation of the serotonergic system in chronic fatigue syndrome. Psychoneuroendocrinology..

[29] Meyer B, Nguyen CB, Moen A, Fagermoen E, Sulheim D, Nilsen H (2015). Maintenance of chronic fatigue syndrome (CFS) in young CFS patients is associated with the 5-HTTLPR and SNP rs25531 A > G Genotype. PLoS ONE.

[30] Sulheim D, Fagermoen E, Winger A, Andersen AM, Godang K, Muller F (2014). Disease mechanisms and clonidine treatment in adolescent chronic fatigue syndrome: a combined cross-sectional and randomized clinical trial. JAMA pediatrics..

[31] Klimas NG, Broderick G, Fletcher MA (2012). Biomarkers for chronic fatigue. Brain Behav Immun.

[32] Montoya JG, Holmes TH, Anderson JN, Maecker HT, Rosenberg-Hasson Y, Valencia IJ (2017). Cytokine signature associated with disease severity in chronic fatigue syndrome patients. Proc Natl Acad Sci USA..

[33] Nguyen CB, Alsøe L, Lindvall JM, Sulheim D, Fagermoen E, Winger A (2017). Whole blood gene expression in adolescent chronic fatigue syndrome: an exploratory cross-sectional study suggesting altered B cell differentiation and survival. J Transl Med..

[34] Lacourt TE, Vichaya EG, Chiu GS, Dantzer R, Heijnen CJ (2018). The high costs of low-grade inflammation: persistent fatigue as a consequence of reduced cellular-energy availability and non-adaptive energy expenditure. Front Behav Neurosci..

[35] Wyller VB, Saul JP, Walløe L, Thaulow E (2008). Sympathetic cardiovascular control during orthostatic stress and isometric exercise in adolescent chronic fatigue syndrome. Eur J Appl Physiol.

[36] Wyller VB, Due R, Saul JP, Amlie JP, Thaulow E (2007). Usefulness of an abnormal cardiovascular response during low-grade head-up tilt-test for discriminating adolescents with chronic fatigue from healthy controls. Am J Cardiol.

[37] Hurum H, Sulheim D, Thaulow E, Wyller VB (2011). Elevated nocturnal blood pressure and heart rate in adolescent chronic fatigue syndrome. Acta Paediatr.

[38] Martínez-Martínez LA, Mora T, Vargas A, Fuentes-Iniestra M, Martínez-Lavín M (2014). Sympathetic nervous system dysfunction in fibromyalgia, chronic fatigue syndrome, irritable bowel syndrome, and interstitial cystitis: a review of case-control studies. J Clin Rheumatol..

[39] Wyller VB, Barbieri R, Thaulow E, Saul JP (2008). Enhanced vagal withdrawal during mild orthostatic stress in adolescents with chronic fatigue. Ann Noninvasive Electrocardiol..

[40] Wyller VB, Godang K, Morkrid L, Saul JP, Thaulow E, Walloe L (2007). Abnormal thermoregulatory responses in adolescents with chronic fatigue syndrome: relation to clinical symptoms. Pediatrics.

[41] Papadopoulos AS, Cleare AJ (2012). Hypothalamic-pituitary-adrenal axis dysfunction in chronic fatigue syndrome. Nat Rev Endocrinol..

[42] Segal TY, Hindmarsh PC, Viner RM (2005). Disturbed adrenal function in adolescents with chronic fatigue syndrome. J Pediatr Endocrinol Metab.

[43] Nijhof SL, Rutten JM, Uiterwaal CS, Bleijenberg G, Kimpen JL, Putte EM (2014). The role of hypocortisolism in chronic fatigue syndrome. Psychoneuroendocrinology..

[44] Roerink ME, Roerink S, Skoluda N, van der Schaaf ME, Hermus A, van der Meer JWM (2018). Hair and salivary cortisol in a cohort of women with chronic fatigue syndrome. Horm Behav.

[45] Wyller VB, Vitelli V, Sulheim D, Fagermoen E, Winger A, Godang K (2016). Altered neuroendocrine control and association to clinical symptoms in adolescent chronic fatigue syndrome: a cross-sectional study. J Transl Med..

[46] Majer M, Welberg LA, Capuron L, Miller AH, Pagnoni G, Reeves WC (2008). Neuropsychological performance in persons with chronic fatigue syndrome: results from a population-based study. Psychosom Med.

[47] Cockshell SJ, Mathias JL (2010). Cognitive functioning in chronic fatigue syndrome: a meta-analysis. Psychol Med.

[48] Thomas M, Smith A (2009). An investigation into the cognitive deficits associated with chronic fatigue syndrome. Open Neurol J..

[49] van de Putte EM, Böcker KB, Buitelaar J, Kenemans JL, Engelbert RH, Kuis W (2008). Deficits of interference control in adolescents with chronic fatigue syndrome. Arch Pediatr Adolesc Med.

[50] Sulheim D, Fagermoen E, Sivertsen OS, Winger A, Wyller VB, Oie MG (2015). Cognitive dysfunction in adolescents with chronic fatigue: a cross-sectional study. Arch Dis Child.

[51] Haig-Ferguson A, Tucker P, Eaton N, Hunt L, Crawley E (2009). Memory and attention problems in children with chronic fatigue syndrome or myalgic encephalopathy. Arch Dis Child.

[52] Kawatani J, Mizuno K, Shiraishi S, Takao M, Joudoi T, Fukuda S (2011). Cognitive dysfunction and mental fatigue in childhood chronic fatigue syndrome a 6-month follow-up study. Brain Dev.

[53] Polli A, Van Oosterwijck J, Meeus M, Lambrecht L, Nijs J, Ickmans K (2019). Exercise-induce hyperalgesia, complement system and elastase activation in Myalgic Encephalomyelitis/chronic fatigue syndrome-a secondary analysis of experimental comparative studies. Scand J Pain..

[54] van de Putte EM, Uiterwaal CS, Bots ML, Kuis W, Kimpen JL, Engelbert RH (2005). Is chronic fatigue syndrome a connective tissue disorder? A cross-sectional study in adolescents. Pediatrics..

[55] Winger A, Kvarstein G, Wyller VB, Sulheim D, Fagermoen E, Småstuen MC (2014). Pain and pressure pain thresholds in adolescents with chronic fatigue syndrome and healthy controls: a cross-sectional study. BMJ Open.

[56] Nijs J, Meeus M, Van Oosterwijck J, Ickmans K, Moorkens G, Hans G (2012). In the mind or in the brain? Scientific evidence for central sensitisation in chronic fatigue syndrome. Eur J Clin Invest.

[57] Washington SD, Rayan RU, Garner R, Provenzano D, Zajur K, Addiego FM, VanMeter JW, Baraniuk JM (2020). Exercise alters brain activation in Gulf War Illness and Myalgic Encephalomyelitis/Chronic Fatigue Syndrome. Brain Commun.

[58] Wyller VB, Sorensen O, Sulheim D, Fagermoen E, Ueland T, Mollnes TE (2015). Plasma cytokine expression in adolescent chronic fatigue syndrome. Brain Behav Immun.

[59] Fagermoen E, Sulheim D, Winger A, Andersen AM, Gjerstad J, Godang K (2015). Effects of low-dose clonidine on cardiovascular and autonomic variables in adolescents with chronic fatigue: a randomized controlled trial. BMC Pediatr..

[60] Fortin J, Habenbacher W, Heller A, Hacker A, Grullenberger R, Innerhofer J (2006). Non-invasive beat-to-beat cardiac output monitoring by an improved method of transthoracic bioimpedance measurement. Comput Biol Med.

[61] Bianchi AM, Mainardi LT, Meloni C, Chierchia S, Cerutti S (1997). Continuous monitoring of the sympatho-vagal balance through spectral analysis. IEEE Eng Med Biol Mag.

[62] Tsunoda M (2006). Recent advances in methods for the analysis of catecholamines and their metabolites. Anal Bioanal Chem.

[63] Gatti R, Antonelli G, Prearo M, Spinella P, Cappellin E, De Palo EF (2009). Cortisol assays and diagnostic laboratory procedures in human biological fluids. Clin Biochem.

[64] Wechler D (2003). Wechsler intelligence scale for children.

[65] Delis DC, Kaplan E, Kramer JH (2001). The Delis–Kaplan Executive Function System (D-KEFS) (Norwegian version).

[66] Benedict RHB, Schretlen D, Groninger L, Brandt J (1998). Hopkins verbal learning test—revised: normative data and analysis of inter-form and test-retest reliability. Clin Neuropsychol..

[67] Matre D, Olsen MB, Jacobsen LM, Klein T, Gjerstad J (2013). Induction of the perceptual correlate of human long-term potentiation (LTP) is associated with the 5-HTT genotype. Brain Res.

[68] Wagner D, Nisenbaum R, Heim C, Jones JF, Unger ER, Reeves WC (2005). Psychometric properties of the CDC Symptom Inventory for assessment of chronic fatigue syndrome. Popul Health Metrics..

[69] Chalder T, Berelowitz G, Pawlikowska T, Watts L, Wessely S, Wright D (1993). Development of a fatigue scale. J Psychosom Res.

[70] Costello EJ, Angold A (1988). Scales to assess child and adolescent depression: checklists, screens, and nets. J Am Acad Child Adolesc Psychiatry.

[71] Varni JW, Seid M, Rode CA (1999). The PedsQL: measurement model for the pediatric quality of life inventory. Med Care.

[72] Walker LS, Greene JW (1991). The functional disability inventory: measuring a neglected dimension of child health status. J Pediatr Psychol.

[73] Grant PM, Ryan CG, Tigbe WW, Granat MH (2006). The validation of a novel activity monitor in the measurement of posture and motion during everyday activities. Br J Sports Med.

[74] Dolnicar S. A Review of Unquestioned Standards in Using Cluster Analysis for Data-Driven Market Segmentation. Wollongong University, Australia, 2002. https://ro.uow.edu.au/commpapers/273.

[75] Vink M, Vink-Niese A (2019). Cognitive behavioural therapy for myalgic encephalomyelitis/chronic fatigue syndrome is not effective. Re-analysis of a Cochrane review. Health Psychol Open..

[76] Kuppuswamy A (2017). The fatigue conundrum. Brain.

[77] Bedree H, Sunnquist M, Jason LA (2019). The DePaul symptom questionnaire-2: a validation study. Fatigue.

