# Supplementary material for: Are there subgroups of chronic fatigue syndrome? An exploratory cluster analysis of biological markers
Source: J Transl Med. 2021 Jan 30;19:48. doi: 10.1186/s12967-021-02713-9 (PMC7847574; doi:10.1186/s12967-021-02713-9)
Supplement: Supplementary file 2 — Additional file 2. Results of hierarchical cluster analyses within each subdomain of variables (immunological, autonomic, neuroendocrine, cognitive and sensory processing functions), as well as associations between clusters and constitutional factors, diagnostic criteria, subjective symptoms and prognosis. [file 12967_2021_2713_MOESM2_ESM.docx]

**Cluster analysis of endocrine variables**

**Supplementary Figure 1. Dendrogram and cluster-solution for endocrine variables**

| **Supplementary Table 1. Endocrine variables used for cluster construction** | | | | |
| --- | --- | --- | --- | --- |
| Cluster | **1** | **2** | **3** | **4** |
| *N* | *73* | *10* | *31* | *2* |
| Plasma norepinephrine – pmol/L, mean (SD) | 1916 (695) | 1903 (689) | 2306 (1001) | 2001 (n.a.) |
| Plasma epinephrine –pmol/L, mean (SD) | 309 (108) | 404 (194) | 397 (238) | 252 (n.a.) |
| Plasma cortisol –  nmol/L, mean (SD) | 403 (144) | 309 (155) | 313 (118) | 407 (n.a.) |
| Plasma ACTH –  pmol/L, mean (SD) | 4.4 (2.2) | 2.9 (1.4) | 3.9 (1.4) | 32 (n.a.) |
| Plasma TSH –  mU/L, mean (SD) | 3.0 (1.1) | 1.8 (0.3) | 2.2 (0.7) | 1.9 (n.a.) |
| Plasma free thyroxine – pmol/L, mean (SD) | 16.3 (1.9) | 14.7 (1.3) | 13.4 (1.2) | 14.5 (n.a.) |
| Serum IGF1 –  pmol/L, mean (SD) | 53.9 (13.1) | 51.3 (11.7) | 47.2 (10.7) | 64 (n.a.) |
| Urine NCR –  pmol/nmol, mean (SD) | 12 (4.6) | 24.3 (17.5) | 13.9 (3.6) | 12.7 (n.a.) |
| Urine ECR –  pmol/nmol, mean (SD) | 1.6 (1.0) | 3.4 (2.1) | 1.3 (0.6) | 1.2 (n.a.) |
| Urine CCR –  nmol/nmol, mean (SD) | 3.5 (1.9) | 15.5 (6.4) | 3.9 (1.9) | 4.1 (n.a.) |
| ACTH = Adrenocorticotropic hormone. TSH = Thyroid-stimulating hormone. IGF=Insuline-like growth factor 1. NCR=Norepinephrine Creatinine Ratio. ECR=Epinephrine Creatine Ratio. CCR = Cortisol Creatinine Ratio. N.a. = not applicable. | | | | |

| **Supplementary Table 2. Background, symptoms and prognostic utility of endocrine clusters** | | | | | |
| --- | --- | --- | --- | --- | --- |
| **Background characteristics** | | | | | |
| Cluster | **1** | **2** | **3** | **4** | **P-value^1^** |
| *N* | *73* | *10* | *31* | *2* |  |
| Sex, nr. of males (%) | 18 (24.7) | 3 (30.0) | 8 (25.8) | 2 (100) | 0.935 |
| Age – years, mean  [95 % CI] | 15.5  [15.2, 15.9] | 15.5  [14.4, 16.6] | 15.0  [14.3, 15.7] | 15.4  [n.a.] | 0.262 |
| BMI, Mean  [95 % CI] | 21.2  [20.4, 22.0] | 25.1  [21.4, 28.8] | 20.9  [19.1, 22.7] | 22.3  [n.a.] | 0.014 |
| Disease duration - months, mean [95 % CI] | 21.2  [18.2, 24.2] | 27.3  [4.0, 50.7] | 18.7  [14.4, 23.0] | 30.0  [n.a.] | 0.290 |
| Canada - no. fulfilling  criteria (%)^2^ | 30 (43.5) | 2 (20.0) | 14 (46.7) | 0 | 0.322 |
|  | | | | | |
| **Symptoms at baseline** | | | | | |
| *N* | *73* | *10* | *31* | *2* |  |
| CFQ, Mean  [95 % CI] | 18.9  [17.4, 20.4] | 18.2  [13.3, 23.1] | 20.2  [18.0, 22.4] | 22.0  [n.a.] | 0.546 |
| PEM, Mean, (Mean Rank) | 3.9 (54.9) | 4.4 (70.9) | 3.9 (51.9) | 3.5 (n.a.) | 0.222 |
| MFQ, Median (IQR) | 17.0 (15.3) | 13.0 (14.3) | 15.0 (13.0) | 18.0 (n.a.) | 0.496 |
| Steps per day, Mean  [95 % CI] | 4204  [3700, 4708] | 5804  [3580, 8029] | 5179  [4309, 6049] | 3228  [n.a.] | 0.036 |
| FDI, Median (IQR) | 23.0 (14.5) | 16.5 (16.3) | 22.0 (13.0) | 27.0 (n.a.) | 0.160 |
| PedsQL, Mean  [95 % CI] | 46.5  [43.3, 49.8] | 54.7  [45.3, 64.1] | 51.8  [48.0, 55.6] | 46.8  [n.a.] | 0.053 |
|  | | | | | |
| **Difference in symptom score week 30 - baseline** | | | | | |
| *N^3^* | *56-59* | *7-8* | *24-26* | *2* |  |
| CFQ, Mean  [95 % CI] | -3.8  [-5.4, -2.1] | -7.6  [-14.7, -0.6] | -7.2  [-10.3, -4.2] | 6.5  [n.a.] | 0.062 |
| PEM, Mean (Mean Rank) | -0.4 (45.5) | -1.1 (36.4) | -0.3 (48.5) | -0.5 (n.a.) | 0.500 |
| MFQ, Mean  [95 % CI] | -1.8  [-3.9, 0.3] | -2.8  [-10.5, 5.0] | -1.3  [-5.0, 2.4] | -2.0  [n.a.] | 0.907 |
| Steps per day, Mean  [95 % CI] | -33  [-731, 665] | -717  [-3167, 1733] | 303  [-688, 1294] | 1621  [n.a.] | 0.626 |
| FDI, Mean  [95 % CI] | -3.2  [-5.8, -0.5] | -6.4  [-17.8, 4.9] | -4.2  [-8.4, -0.1] | -3.5  [n.a.] | 0.694 |
| PedsQL, Mean  [95 % CI] | 5.6  [2.1, 9.1] | 11.2  [-4.9, 27.2] | 6.2  [0.5, 11.9] | 12.0  [n.a.] | 0.583 |
| BMI=Body Mass Index. CFQ=Chalder Fatigue Questionnaire. PEM=Post Exertional Malaise. MFQ=Mood and Fatigue Questionnaire. FDI=Functional disability inventory. PedsQL=The Pediatric Quality of Life Inventory. CI=Confidence Interval. IQR=Interquartile range. ^1^P-values not corrected for multiple tests. The p-values are based on one-way ANOVA, Kruskal-Wallis test or Fisher Exact Test as appropriate. Only group 1-3 were used in the statistical analyses due to few participants in group 4. ^2^Number of patients fulfilling the Canada 2003 diagnostic criteria for Chronic Fatigue Syndrome [Carruthers 2003].^3^N varies due to missing data. | | | | | |

**Supplementary Figure 2. Scatterplots of important variables in constructing endocrine cluster**

**Cluster analysis of inflammation variables**

**Supplementary Figure 3. Dendrogram and cluster-solution for inflammation variables**

| **Supplementary Table 3. Inflammation variables used for cluster construction** | | | | |
| --- | --- | --- | --- | --- |
| Cluster | **1** | **2** | **3** | **4** |
| *N* | *59* | *14* | *36* | *7* |
| Plasma high sensitivite CRP – mg/L, median (IQR) | 0.36 (0.24, 0.82) | 0.44 (0.29, 1.28) | 0.53 (0.23, 1.13) | 7.2 (1.3, 14.0) |
| TCC –  CAU/mL, mean (SD) | 0.68 (0.24) | 0.57 (0.16) | 0.63 (0.23) | 1.07 (0.89) |
| Interferon gamma –  pg/mL, median (IQR) | 71.6 (52, 119) | 247 (161, 269) | 92 (48, 120) | 142 (59, 207) |
| MCP-1 –  pg/mL, mean (SD) | 9.1 (4.8) | 21.3 (7.0) | 11.4 (4.0) | 13.7 (6.6) |
| IP-10^7^ –  pg/mL, median (IQR) | 316 (196, 457) | 337 (289, 620) | 325 (244, 444) | 2240 (1053, 2753) |
| Blood leucocytes –  10^9^ cells/L, mean (SD) | 5.1 (1.0) | 6.7 (1.3) | 6.8 (1.4) | 7.0 (1.6) |
| Blood lymphocytes –  10^9^ cells/L, mean (SD) | 1.9 (0.4) | 2.9 (0.6) | 2.5 (0.5) | 2.3 (1.2) |
| Blood eosinophils –  10^9^ cells/L, mean (SD) | 0.12 (0.06) | 0.18 (0.11) | 0.29 (0.14) | 0.16 (0.15) |
| CRP=C-reactive protein. IQR=Interquartile Range. SD=Standard Deviation. TCC=Terminal Complement Complex. MCP=Monocyte Chemotactic Protein. IP=Interferon gamma induced protein. | | | | |

| **Supplementary Table 4. Background, symptoms and prognostic utility of inflammation clusters** | | | | | |
| --- | --- | --- | --- | --- | --- |
| **Background characteristics** | | | | | |
| Cluster | **1** | **2** | **3** | **4** | **P-value^1^** |
| *N* | *59* | *14* | *36* | *7* |  |
| Sex, nr. of males (%) | 17 (28.8) | 5 (35.7) | 8 (22.8) | 1 (14.3) | 0.696 |
| Age – years, mean  [95 % CI] | 15.4  [15.0, 15.8] | 15.1  [14.2, 16.0] | 15.4  [14.8, 16.1] | 15.7  [14.1, 17.3] | 0.863 |
| BMI, Median  (IQR) | 20.9  (18.3, 23.1) | 20.0  (17.3, 23.8) | 22.6  (18.9, 25.9) | 19.6  (16.0, 23.1) | 0.354 |
| Disease duration - months, median (IQR) | 18.0  (12.0, 27.0) | 13.0  (8.0, 18.5) | 24.0  (9.8, 28.5) | 21.0  (13.0, 24.0) | 0.301 |
| Canada - no. fulfilling  criteria (%)^2^ | 24 (42.1) | 7 (53.8) | 12 (42.9) | 3 (42.9) | 0.704 |
|  | | | | | |
| **Symptoms at baseline** | | | | | |
| *N* | *59* | *14* | *36* | *7* |  |
| CFQ, Mean  [95 % CI] | 19.0  [17.3, 20.7] | 20.2  [16.7, 23.6] | 18.9  [16.8, 21.0] | 21.4  [15.9, 27.0] | 0.716 |
| PEM, Mean, (Mean Rank) | 3.9 (55.8) | 4.1 (59.8) | 4.0 (57.5) | 3.9 (50.8) | 0.930 |
| MFQ, Median  (IQR) | 15.0  (10.0, 22.0) | 16.0  (6.5, 24.5) | 17.5  (10.0, 24.5) | 24.0  (13.0, 27.0) | 0.798 |
| Steps per day, Mean  [95 % CI] | 4549  [3919, 5179] | 4729  [3193, 6265] | 4565  [3836, 5294] | 4769  [2372, 7166] | 0.991 |
| FDI, Mean  [95 % CI] | 23.6  [20.9, 26.2] | 25.5  [20.7, 30.6] | 22.6  [19.7, 25.6] | 25.1  [16.5, 33.8] | 0.763 |
| PedsQL, Mean  [95 % CI] | 48.0  [44.6, 51.3] | 45.2  [36.0, 54.4] | 51.3  [46.8, 55.7] | 48.0  [40.8, 55.1] | 0.469 |
|  | | | | | |
| **Difference in symptom score week 30 - baseline** | | | | | |
| *N^3^* | *42-46* | *11-13* | *30-31* | *6* |  |
| CFQ, Mean  [95 % CI] | -4.4  [-6.7, -2.1] | -4.3  [-7.1, -1.4] | -6.0  [-8.7, -3.3] | -8.3  [-14.9, -1.8] | 0.515 |
| PEM, Mean  [95 % CI] | -0.7  [-1.1, -0.3) | 0.0  [-0.8, 0.8] | -0.1  [-0.4, 0.2] | -1.2  [-2.4, 0.1] | 0.056 |
| MFQ, Mean  [95 % CI] | -1.1  [-4.0, 1.8] | -1.7  [-4.8, 1.4] | -2.2  [-4.9, 0.5] | -4.2  [-12.6, 4.2] | 0.831 |
| Steps per day, Mean  [95 % CI] | -90  [-824, 643] | -241  [-1478, 996] | 193  [-828, 1214] | 823  [-3658, 5305] | 0.828 |
| FDI, Mean  [95 % CI] | -3.0  [-6.3, 0.4] | -6.3  [-9.6, -2.9] | -2.9  [-6.2, 0.3] | -7.7  [-24.7, 9.4] | 0.550 |
| PedsQL, Mean  [95 % CI] | 8.7  [4.0, 13.4] | 7.2  [-3.3, 17.7] | 2.9  [-1.2, 7.1] | 6.0  [1.8, 10.2] | 0.369 |
| BMI=Body Mass Index. CFQ=Chalder Fatigue Questionnaire. PEM=Post Exertional Malaise. MFQ=Mood and Fatigue Questionnaire. FDI=Functional disability inventory. PedsQL=The Pediatric Quality of Life Inventory. CI=Confidence Interval. IQR=Interquartile range. ^1^P-values not corrected for multiple tests. The p-values are based on one-way ANOVA, Kruskal-Wallis test or Fisher Exact Test as appropriate. ^2^Number of patients fulfilling the Canada 2003 diagnostic criteria for Chronic Fatigue Syndrome [Carruthers 2003].^3^N varies due to missing data. | | | | | |

**Supplementary Figure 4. Scatterplots of important variables in constructing inflammation cluster**

*Squared transformation is performed to achieve normal distribution suitable for graphical evaluation.*

**Cluster analysis of cardiovascular variables**

**Supplementary Figure 5. Dendrogram and cluster-solution for cardiovascular variables**

| **Supplementary Table 5. Cardiovascular variables used for cluster construction** | | | |
| --- | --- | --- | --- |
| Cluster | **1** | **2** | **3** |
| *N* | *24* | *60* | *32* |
| Heart Rate supine – beats/min, mean (SD) | 66.9 (8.6) | 66.8 (7.6) | 82.6 (8.8) |
| SBP supine, mmHg,  mean (SD) | 104.7 (11.5) | 103.6 (9.9) | 108.2 (9.0) |
| Stroke Volum Index – mL/BSA,  mean (SD) | 49.8 (6.7) | 47.9 (8.6) | 40.0 (7.5) |
| TPR-index – TPR/BSA,  mean x 10^3^ (SD) | 8.0 (1.7) | 9.2 (2.1) | 9.6 (1.5) |
| RR variability HF power – ms^2^, median (IQR) | 1737 (846, 2956) | 2126 (602, 2550) | 153 (100, 422) |
| SBP variability LF power - ms^2^, median (IQR) | 2.6 (1.4, 6.0) | 4.0 (2.1, 7.1) | 2.7 (1.4, 4.4) |
| Ratio LF/HF-RR-interval,  median (IQR) | 0.64 (0.31, 0.81) | 0.53 (0.38, 0.84) | 1.46 (1.01, 1.89 ) |
| Heart rate difference^1^ – beats/min, mean (SD) | 9.2 (4.7) | 2.7 (2.7) | 6.3 (3.6) |
| SBP difference^1^ – mmHg,  mean (SD) | 2.1 (4.9) | 0.7 (3.9) | -1.7 (3.8) |
| DBP difference ^1^ – mmHg,  mean (SD) | 5.0 (3.3) | 1.1 (2.5) | -0.1 (2.3) |
| SV- Index difference^1^ – mL/BSA, mean (SD) | -10.6 (4.7) | -4.1 (3.1) | -4.1 (4.8) |
| Ratio LF/HF-RR-interval difference^1^, mean (SD) | 0.57 (0.52) | 0.13 (0.33) | 0.75 (1.13) |
| SBP=Systolic Blood Pressure. DBP=Diastolic Blood Pressure. SV=Stroke Volume. BSA=Body Surface Area; Du Bois-formula. TRP=Total Peripheral Resistance. HF=High Frequency. LF=Low Frequency. SD=Standard Deviation. IQR=Interquartile Range. ^1^Difference between supine position and after 20-degree head-up-tilt. | | | |

| **Supplementary Table 6. Background, symptoms and prognostic utility of cardiovascular clusters** | | | | |
| --- | --- | --- | --- | --- |
| **Background characteristics** | | | | |
| Cluster | **1** | **2** | **3** | **P-value^1^** |
| *N* | *24* | *60* | *32* |  |
| Sex, nr. of males (%) | 11 (45.8) | 15 (25.0) | 5 (15.6) | 0.045 |
| Age – years, mean  [95 % CI] | 15.4  [14.9, 16.0] | 15.6  [15.2, 16.0] | 14.8  [14.3, 15.4] | 0.062 |
| BMI, Mean  [95 % CI] | 22.0  [20.0, 24.0] | 21.4  [20.4, 22.4] | 21.3  [19.5, 22.8] | 0.786 |
| Disease duration - months, median (IQR) | 16.0  (10.3, 24.8) | 18.0  (12.0, 26.0) | 17.0  (11.8, 28.0) | 0.704 |
| Canada - no. fulfilling  criteria (%)^2^ | 10 (41.7) | 21 (38.2) | 15 (46.9) | 0.743 |
|  | | | | |
| **Symptoms at baseline** | | | | |
| *N* | *24* | *60* | *32* |  |
| CFQ, Mean  [95 % CI] | 20.1  [18.0, 22.3] | 18.5  [16.8, 20.2] | 19.9  [17.7, 22.2] | 0.435 |
| PEM, Mean, (Mean Rank) | 3.9 (53.6) | 4.1 (54.7) | 4.0 (61.9) | 0.506 |
| MFQ, Median (IQR) | 15.9 (7.3, 22.8) | 17.3 (9.5, 23.5) | 19.1 (12.5, 25.8) | 0.486 |
| Steps per day, Mean  [95 % CI] | 4706  [3696, 5717] | 4895  [4293, 5496] | 3906  [3085, 4728] | 0.155 |
| FDI, Mean  [95 % CI] | 20.0 (6.5) | 22.8 (8.5) | 27.6 (10.6) | 0.005 |
| PedsQL, Mean  [95 % CI] | 49.9  [45.6, 54.2] | 50.0  [46.5, 53.6] | 45.4  [40.6, 50.3] | 0.240 |
|  | | | | |
| **Difference in symptom score week 30 - baseline** | | | | |
| *N^3^* | *20-22* | *45-47* | *26* |  |
| CFQ, Mean  [95 % CI] | -8.8  [-12.3, -5.2] | -3.8  [-5.9, -1.7] | -4.7  [-7.1, -2.2] | 0.030 |
| PEM, Mean  [95 % CI] | -0.6  [-1.2, 0.0] | -0.5  [-0.9, -0.2] | -0.3  [-0.6, 0.2] | 0.605 |
| MFQ, Mean  [95 % CI] | -3.6  [-6.6, -0.6] | -0.4  [-2.9, 2.2] | -2.6  [-6.2, 0.9] | 0.273 |
| Steps per day, Mean  [95 % CI] | 208  [-804, 1220] | 355  [-504, 1214] | -685  [-1559, 188] | 0.250 |
| FDI, Mean  [95 % CI] | -4.3  [-9.1, 0.6] | -4.0  [-6.6, -1.4] | -2.8  [-7.5, 2.0] | 0.851 |
| PedsQL, Mean  [95 % CI] | 11.3  [4.5, 18.1] | 6.1  [1.5, 10.8] | 2.7  [-0.3, 5.7] | 0.105 |
| BMI=Body Mass Index. CFQ=Chalder Fatigue Questionnaire. PEM=Post Exertional Malaise. MFQ=Mood and Fatigue Questionnaire. FDI=Functional disability inventory. PedsQL=The Pediatric Quality of Life Inventory. CI=Confidence Interval. IQR=Interquartile range. ^1^P-values not corrected for multiple tests. The p-values are based on one-way ANOVA, Kruskal-Wallis test or Fisher Exact Test as appropriate. ^2^Number of patients fulfilling the Canada 2003 diagnostic criteria for Chronic Fatigue Syndrome [Carruthers 2003].^3^N varies due to missing data. | | | | |

**Supplementary Figure 6. Scatterplots of important variables in constructing cardiovascular cluster**

*Ln-transformation is performed to achieve normal distribution suitable for graphical evaluation.*

**Cluster analysis of pressure pain threshold variable (PPT)**

**Supplementary Figure 7. Dendrogram and cluster-solution for PPT-variable**

| **Supplementary Table 7. PPT variable used for cluster construction** | | | |
| --- | --- | --- | --- |
| Cluster | **1** | **2** | **3** |
| *N* | *17* | *47* | *51* |
| Pressure pain threshold trapezius – Newton,  mean (SD) | 28.5 (6.1) | 9.2 (2.3) | 17.0 (2.7) |
| SD=Standard Deviation. | | | |

| **Supplementary Table 8. Background, symptoms and prognostic utility of PPT clusters** | | | | |
| --- | --- | --- | --- | --- |
| **Background characteristics** | | | | |
| Cluster | **1** | **2** | **3** | **P-value^1^** |
| *N* | *17* | *47* | *51* |  |
| Sex, nr. of males (%) | 2 (11.8) | 15 (31.9) | 13 (25.5) | 0.290 |
| Age – years, mean  [95 % CI] | 16.2  [15.6, 16.8] | 15.0  [14.5, 15.5] | 15.5  [15.0, 15.9] | 0.018 |
| BMI, Mean  [95 % CI] | 22.3  [20.9, 23.7] | 21.3  [19.8, 22.8] | 21.3  [20.2, 22.3] | 0.665 |
| Disease duration - months, median (IQR) | 18  (7.0, 24.0) | 18  (9.5, 26.3) | 19  (12.0, 26.5) | 0.567 |
| Canada - no. fulfilling  criteria (%)^2^ | 7 (46.7) | 25 (55.6) | 14 (28.0) | 0.020 |
|  | | | | |
| **Symptoms at baseline** | | | | |
| *N* | *17* | *47* | *51* |  |
| CFQ, Mean  [95 % CI] | 18.9  [16.0, 21.7] | 20.1  [18.3, 22.0] | 18.7  [17.0, 20.5] | 0.516 |
| PEM, Mean, (Mean Rank) | 4.1 (59.1) | 4.1 (61.1) | 3.8 (50.4) | 0.214 |
| MFQ, Median  (IQR) | 19.6  (16.0, 23.8) | 18.5  (10.0, 25.3) | 16.2  (8.0, 22.3) | 0.246 |
| Steps per day, Mean  [95 % CI] | 3965  [3211, 4718] | 4353  [3681, 5025] | 5013  [4280, 5746] | 0.190 |
| FDI, Mean  [95 % CI] | 22.8  [19.9, 25.6] | 26.8  [23.7, 29.8] | 21.1  [18.7, 23.5] | 0.009 |
| PedsQL, Mean  [95 % CI] | 46.1  [40.7, 51.6] | 44.9  [41.4, 48.5] | 52.9  [49.0, 56.7] | 0.006 |
|  | | | | |
| **Difference in symptom score week 30 - baseline** | | | | |
| *N^3^* | *13-15* | *37-38* | *40-42* |  |
| CFQ, Mean  [95 % CI] | -3.1  [-6.4, 0.1] | -5.7  [-7.9, -3.5] | -5.3  [-7.8, -2.8] | 0.509 |
| PEM, Mean  [95 % CI] | -0.3  [-1.1, 0.5] | -0.6  [-1.0, -0.2] | -0.4  [-0.7, 0.0] | 0.548 |
| MFQ, Median  (IQR) | -2.0  (-8.0, 2.0) | -3.0  (-10.0, 5.3) | 0.0  (-4.5, 4.0) | 0.270 |
| Steps per day, Mean  [95 % CI] | 85  [-1025, 1195] | -18  [-674, 639] | 68  [-947, 1083] | 0.987 |
| FDI, Mean  [95 % CI] | -2.3 (8.7) | -5.7 (10.7) | -2.2 (9.5) | 0.259 |
| PedsQL, Mean  [95 % CI] | 6.7 (13.3) | 7.8 (14.0) | 5.1 (14.4) | 0.695 |
| PPT=Pressure Pain Threshold. BMI=Body Mass Index. CFQ=Chalder Fatigue Questionnaire. PEM=Post Exertional Malaise. MFQ=Mood and Fatigue Questionnaire. FDI=Functional disability inventory. PedsQL=The Pediatric Quality of Life Inventory. CI=Confidence Interval. IQR=Interquartile range. ^1^P-values not corrected for multiple tests. The p-values are based on one-way ANOVA, Kruskal-Wallis test or Fisher Exact Test as appropriate. ^2^Number of patients fulfilling the Canada 2003 diagnostic criteria for Chronic Fatigue Syndrome [Carruthers 2003].^3^N varies due to missing data. | | | | |

**Supplementary Figure 8. Scatterplots of important variables in constructing PPT clusters**

**Cluster analysis of cognitive variables**

**Supplementary Figure 9. Dendrogram and cluster-solution for cognitive variables**

| **Supplementary Table 9. Cognitive variables used for cluster construction** | | |
| --- | --- | --- |
| Cluster | **1** | **2** |
| *N* | *45* | *71* |
| Digit span forward^1^ – total score,  mean (SD) | 7.0 (1.1) | 9.2 (1.9) |
| Digit span backward^1^, total score,  mean (SD) | 4.8 (1.3) | 6.4 (2.0) |
| Color-Word interference test^2^, condition 2 – sec, mean (SD) | 31.0 (7.6) | 23.9 (3.8) |
| Color-Word interference test^2^, condition 3 – sec,  mean (SD) | 71.6 (16.3) | 52.1 (8.0) |
| Color-Word interference test^2^, condition 4 – sec,  mean (SD) | 78.6 (15.7) | 60.2 (10.7) |
| Verbal learning^3^ – total sum across three tests,  mean (SD) | 25.0 (4.0) | 28.5 (3.4) |
| ^1^From WISC-IV test battery ^2^From the D-KEFS test battery ^3^From the HVLT test battery | | |

| **Supplementary Table 10. Background, symptoms and prognostic utility of cognitive clusters** | | | |
| --- | --- | --- | --- |
| **Background characteristics** | | | |
| Cluster | **1** | **2** | **P-value^1^** |
| *N* | *45* | *71* |  |
| Sex, nr. of males (%) | 12 (26.7) | 19 (26.8) | 1.000 |
| Age – years, mean  [95 % CI] | 14.7  [14.2, 15.2] | 15.8  [15.5, 16.2] | >0.001 |
| BMI, Mean  [95 % CI] | 20.9  [19.3, 22.3] | 21.9  [21.0, 22.8] | 0.203 |
| Disease duration - months, median (IQR) | 18.0  (8.0, 24.0) | 18.0  (12.0, 30.0) | 0.059 |
| Canada - no. fulfilling  criteria (%)^2^ | 22 (51.2) | 24 (35.3) | 0.116 |
|  | | | |
| **Symptoms at baseline** | | | |
| *N* | *45* | *71* |  |
| CFQ, Mean  [95 % CI] | 20.9  [18.9, 22.9] | 18.2  [16.9, 19.6] | 0.023 |
| PEM, Mean, (Mean Rank) | 4.2 (62.9) | 3.8 (52.5) | 0.082 |
| MFQ, Median  (IQR) | 18.6  (8.0, 26.0) | 16.9  (10.0, 22.0) | 0.709 |
| Steps per day, Mean  [95 % CI] | 4625  [3916, 5335] | 4567  [4009, 5125] | 0.897 |
| FDI, Mean  [95 % CI] | 25.5  [22.5, 28.5] | 22.4  [20.3, 24.4] | 0.076 |
| PedsQL, Mean  [95 % CI] | 46.6  [3.9, 4.4] | 50.0  [3.6, 4.1] | 0.181 |
|  | | | |
| **Difference in symptom score week 30 - baseline** | | | |
| *N^3^* | *31-34* | *58-61* |  |
| CFQ, Mean  [95 % CI] | -4.7  [-7.0, -2.3] | -5.4  [-7.3, -3.5] | 0.633 |
| PEM, Mean  [95 % CI] | -0.5  [-1.0, -0.1] | -0.4  [-0.7, -0.1] | 0.993 |
| MFQ, Mean  [95 % CI] | -2.4  [-5.6, 0.8] | -1.4  [-3.4, 0.7] | 0.571 |
| Steps per day, Mean  [95 % CI] | 187  [-689, 1063] | -48  [-733, 638] | 0.676 |
| FDI, Mean  [95 % CI] | -5.0  [-8.3, -1.7] | -2.9  [-5.7, -0.2] | 0.340 |
| PedsQL, Mean  [95 % CI] | 5.7  [1.7, 9.7] | 6.8  [2.8, 10.7] | 0.730 |
| BMI=Body Mass Index. CFQ=Chalder Fatigue Questionnaire. PEM=Post Exertional Malaise. MFQ=Mood and Fatigue Questionnaire. FDI=Functional disability inventory. PedsQL=The Pediatric Quality of Life Inventory. CI=Confidence Interval. IQR=Interquartile range. ^1^P-values not corrected for multiple tests. The p-values are based on one-way ANOVA, Kruskal-Wallis test or Fisher Exact Test as appropriate. ^2^Number of patients fulfilling the Canada 2003 diagnostic criteria for Chronic Fatigue Syndrome [Carruthers 2003].^3^N varies due to missing data. | | | |

**Supplementary Figure 10. Scatterplots of important variables in constructing cognitive clusters**
